# Head and Neck Veins of the Mouse. A Magnetic Resonance, Micro Computed Tomography and High Frequency Color Doppler Ultrasound Study

**DOI:** 10.1371/journal.pone.0129912

**Published:** 2015-06-11

**Authors:** Marcello Mancini, Adelaide Greco, Enrico Tedeschi, Giuseppe Palma, Monica Ragucci, Maria Grazia Bruzzone, Anna Rita Daniela Coda, Enza Torino, Alessandro Scotti, Ileana Zucca, Marco Salvatore

**Affiliations:** 1 Institute of Biostructures and Bioimaging, National Research Council, Naples, Italy; 2 Department of Advanced Biomedical Sciences, University “Federico II”, Naples, Italy; 3 CEINGE-Biotecnologie Avanzate, Naples, Italy; 4 Unit of Neuroradiology, IRCCS Foundation “Carlo Besta” Neurological Institute, Milan, Italy; 5 Center for Advanced Biomaterials for Health Care@CRIB, Istituto Italiano di Tecnologia, Naples, Italy; 6 Scientific Direction, IRCCS Foundation "Carlo Besta" Neurological Institute, Milan, Italy; 7 IRCCS SDN, Naples, Italy; University of Louisville, UNITED STATES

## Abstract

To characterize the anatomy of the venous outflow of the mouse brain using different imaging techniques. Ten C57/black male mice (age range: 7-8 weeks) were imaged with high-frequency Ultrasound, Magnetic Resonance Angiography and ex-vivo Microcomputed tomography of the head and neck. Under general anesthesia, Ultrasound of neck veins was performed with a 20MHz transducer; head and neck Magnetic Resonance Angiography data were collected on 9.4T or 7T scanners, and ex-vivo Microcomputed tomography angiography was obtained by filling the vessels with a radiopaque inert silicone rubber compound. All procedures were approved by the local ethical committee. The dorsal intracranial venous system is quite similar in mice and humans. Instead, the mouse Internal Jugular Veins are tiny vessels receiving the sigmoid sinuses and tributaries from cerebellum, occipital lobe and midbrain, while the majority of the cerebral blood, i.e. from the olfactory bulbs and fronto-parietal lobes, is apparently drained through skull base connections into the External Jugular Vein. Three main intra-extracranial anastomoses, absent in humans, are: 1) the petrosquamous sinus, draining into the posterior facial vein, 2) the veins of the olfactory bulb, draining into the superficial temporal vein through a foramen of the frontal bone 3) the cavernous sinus, draining in the External Jugular Vein through a foramen of the sphenoid bone. The anatomical structure of the mouse cranial venous outflow as depicted by Ultrasound, Microcomputed tomography and Magnetic Resonance Angiography is different from humans, with multiple connections between intra- and extra- cranial veins.

## Introduction

The malfunction of the venous cerebral system can lead to morbidity and could be a cofactor in several neurological diseases [1–12]. Recently, vascular abnormalities in Multiple Sclerosis have been suggested as a possible contributing factor [12, 13]. Therefore, it would be useful to have animal models to study the relationships between the status of venous cerebral district and neurological diseases. Unfortunately, to date, the majority of the studies on mouse cerebral vasculature focused on the arteries and no study of the venous system has been reported.

Due to the high complexity of the cerebral vasculature and the difficulty in obtaining high-resolution in vivo imaging of cerebral mice vessels, the study of this anatomy is a challenging task.

The aim of the present study was to characterize the anatomy of the venous outflow of the mouse brain using different imaging techniques. As a reference work, the mouse cerebrovascular atlas by Dorr et al. [14] was used.

## Material and Methods

### Animal preparation

Animal studies were performed in accordance with National Institutes of Health (NIH) recommendations and Animal Research Advisory Committee (ARAC) procedure [15], after approval by the National Institutional animal research committee (Institutional Animal and Care Committee of the University of Naples “Federico II” and the Italian Ministry of Health). All animal procedures in this study were conducted by a veterinarian and conformed to all regulations protecting animals used for research purposes, including national guidelines and implementation of 2010/63/EU Directive on the protection of animals used for scientific purposes.

Ten C57/black male mice (Charles River Laboratory, Wilmington, MA), aging between 7 and 8 weeks were used for in vivo studies. Body weight range of animals was 25 to 30 gr. Five mice were studied with Ultrasound and microcomputed tomography (micro-CT), three mice with Magnetic Resonance Angiography (MRA) and US, and two mice with MRA only.

Mice were deeply anesthetized using isoflurane (5% induction dose and 2.5% maintenance dose) vaporized in oxygen (2 L/min) on a heated stage with a constant monitoring of their body temperature, using a physiological monitoring platform.

At the end of the experimental procedures mice were euthanised with a CO2 overdose.

### High Frequency Ultrasound with Color Doppler

Under general anesthesia, color flow Doppler sonography was performed to provide a general overview of the neck veins. Before ultrasound, examination hairs were removed from the neck and thorax with a depilatory cream to obtain a direct contact of the ultrasound gel to the skin of the animal minimizing ultrasound attenuation. To provide a coupling medium for the transducer warm gel was used. An outer ring of thick gel (Aquasonic 100; Parker Laboratories, Orange, NJ) was filled with a thinner gel (echo Gel 100; Eco-Med Pharmaceutical, Mississauga, Ontario, Canada) over the region of interest.

Precise and repeatable control over the position of the two-dimensional image plane was obtained with a rail system (Vevo Integrated Rail System II; Visualsonics, Canada). High Frequency Ultrasound was performed with Vevo 2100 (Visualsonics, Canada) and a 20 MHz MicroScan transducer (MS250; Visualsonics, Canada) was used (bandwidth 13–24 MHz, axial resolution 75 μm, lateral resolution 165 μm, color Doppler frequency 16 or 21MHz, Pulsed Doppler frequency 16 MHz, minimum sample volume 0,19 mm). The veins were tracked in the anterior segment of the neck, the blood velocity was determined positioning the cursor placement depth at center of the vessel, the Doppler angle of insonation was maintained below 60°, and samples size was always kept as small as possible. Five readings were averaged, for the pulsatile waveform the lower velocity (EDV) the mean and peak velocity (PSV) and pulsatility index were measured (PI = systolic diastolic/mean)

### Magnetic Resonance Angiography

Magnetic Resonance data were collected on 9.4 T or 7 T Bruker Biospec (Ettlingen, Germany) scanners with a volume transmitter coil and a four-channel rat brain receiver coil. For a global coverage of the whole brain and neck, time-of-flight (TOF) Magnetic Resonance Angiography (MRA) was performed using two multi-slice 2D axial spoiled gradient echo (FLASH) sequences acquired with a flip angle of 80°, a repetition time of 11.8 ms (9.4T) or 19.14ms (7T) and an echo time of 3.9 ms (9.4T) or 4.68 ms (7T) and 8 averages. A field-of-view of 20x20x18.7 mm3 has been acquired with an in-plane resolution of 52 μm (pixel bandwidth: 233 Hz) and 85 slices of 300 μm thickness (slice overlap: 27%).

Each MRA sequence was repeated twice, once with a saturation band positioned at a the thoracic outlet level to null the signal coming from protons in the arterial vessels, and once without the band, thereby displaying the flow signal from both arteries and veins of the head and neck district.

### Micro silicon-CT imaging

Ex vivo micro-CT vessels imaging requires the filling of vessels with a radiopaque compound. An inert silicone rubber compound (MICROFIL, FlowTech Inc., MA) with a low viscosity was perfused in mice to have a complete filling of the cerebral and neck vasculature [16, 17]. In anesthetized mice, an incision was done through the midline of the sternum and the heart was exposed. A 24-gauge IV catheter was connected to a digital peristaltic pump (Peri-Star Pro, 2 channel, High rate, large tubing 220V, WPI Word Precision Instruments, Inc.) and inserted into the left ventricle of the heart and a cut over the right atrium is done. Through an incision on the right atrium the blood in vessels was drained out and then replaced by warm heparinized (5 U/mL) phosphate buffered saline for 5 minutes. The Microfil (1:2 mixture of Microfil to diluent and 5% (v/v) curring agent) was perfused for about 2 min to allow the Microfil to circulate through the cerebral vascular system and was allowed to cure at room temperature for 90 minutes. Microcomputed tomography (Micro-CT) was performed with the scanner Explore Locus (GE Healthcare, Manchester, UK) using the following parameters: 80K X-ray tube voltage, 450 μA X-ray tube current, scan technique 360°. A 160 minutes scan with a spatial resolution of 27 μm was performed. After reconstruction, images were calibrated in Hounsfield units (HU). Qualitative analysis was performed to obtain a tridimensional representation of the cerebral and neck vasculature [18].

## Results

The study of cerebral outflow showed a number of large veins and sinuses that were visible with the different imaging techniques with some differences compared to humans. The cerebral venous outflow of the mouse consists of three paired extracranial districts: external jugular veins (EJVs), internal jugular veins (IJVs) and vertebral veins (VVs).

Some minor caliber asymmetry between left and right vessels (e.g. IJVs or TSs) was observed in our small mouse sample. Apart from that, no remarkable venous variation was found.

### Dorsal and intra-cerebral veins

**Fig 1:**
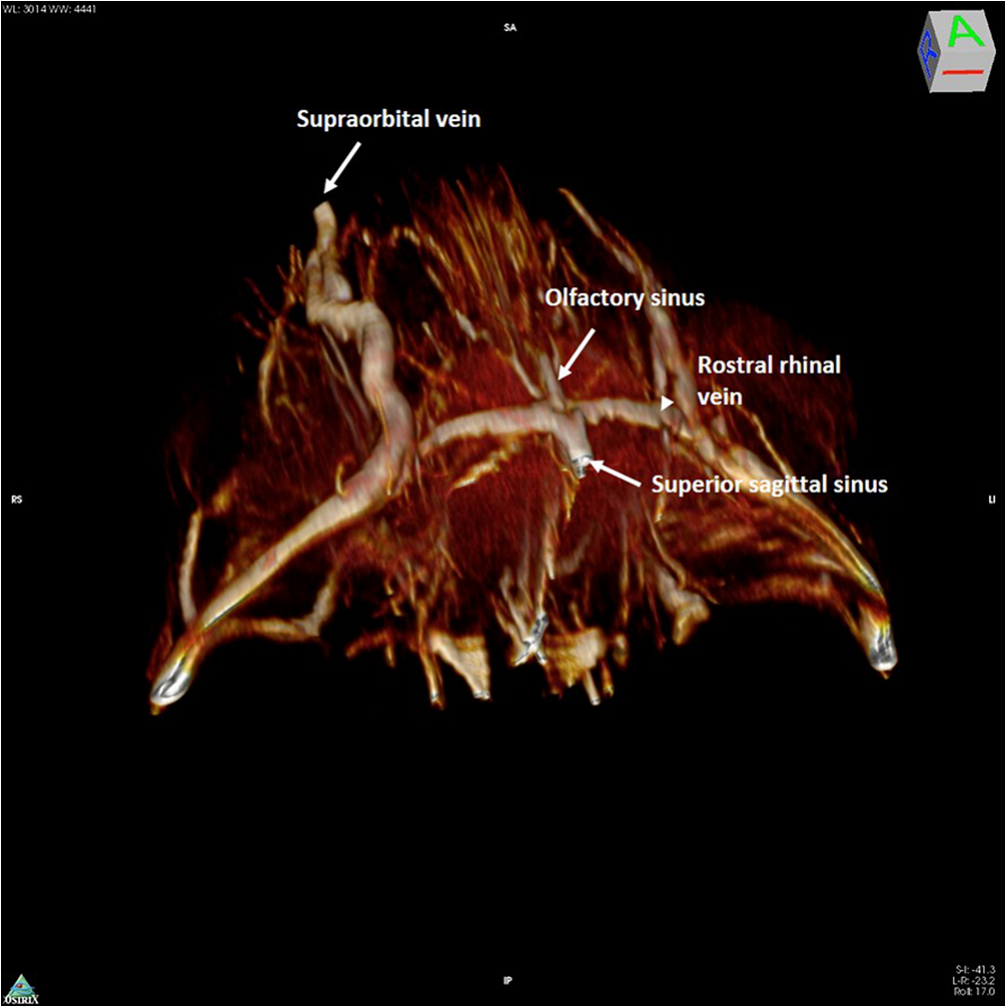
3D rendering of magnetic resonance angiography acquisition of the rostral part of the mouse head. The mouse head is seen from above (the back of the head has been removed in post-processing). The anterior confluence of the median olfactory sinus (thin arrow) with the paired rostral rhinal veins (arrowhead) and the beginning of the superior sagittal sinus (thick arrow) is shown. Extracranially the supraorbital vein is visible.

Intracranially, the most anterior vein visible was a single, median straight vessel, that may be termed the “olfactory sinus”, as it courses dorsally in the olfactory sulcus. After a short rectilinear course, it joins the rostral rhinal veins that form a ring around the olfactory bulbs (Fig 1).

**Fig 2:**
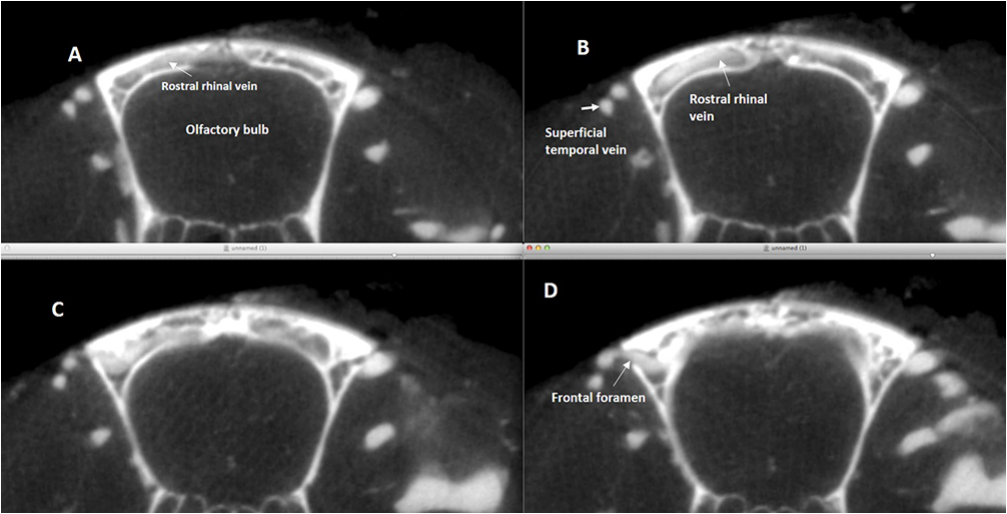
Axial silicon-microcomputed tomography of the rostral rhinal vein. **(A-D)** Four consecutive axial silicon- microcomputed tomography slices in cranio-caudal sequence showing the drainage of each rostral rhinal vein, which, after an intraosseus course, joins with the ipsilateral superficial temporal vein, through the frontal foramen.

**Fig 3:**
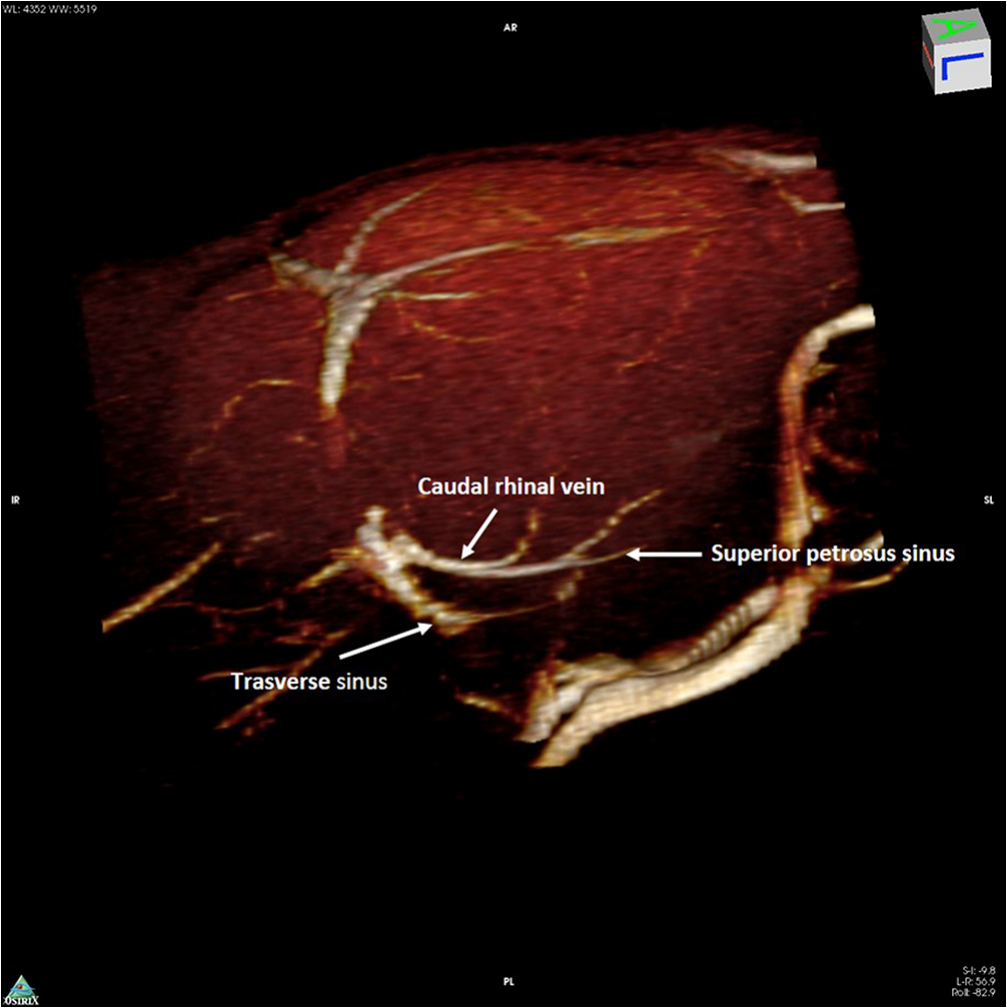
3D rendering of magnetic resonance angiography acquisition of the mouse head. 3D rendering of magnetic resonance angiography acquisition of the caudal part of the mouse head, as seen laterally from a postero-superior angle. The confluence of the left caudal rhinal vein with the ipsilateral transverse sinus, as well as the junction of the superior petrosus sinus, are shown.

These veins originate at the dorsal margin of the olfactory bulbs, extend forward, turn and run inferiorly following the border between the olfactory bulbs and the frontal lobes. These veins drain into the anterior portion of the superior sagittal sinus (SSS) and connect laterally with the superficial temporal vein that begins superficially at the medial corner of the eye as the angular vein and supraorbital vein (Fig 2). The caudal rhinal veins lie in the lateral section of the temporal–parietal lobe and occipital lobes, draining the lateral aspect of these cortices. Posteriorly, the veins join the Transverse sinus (TS), where the occipital lobe meets the cerebellum (Fig 3).

**Fig 4:**
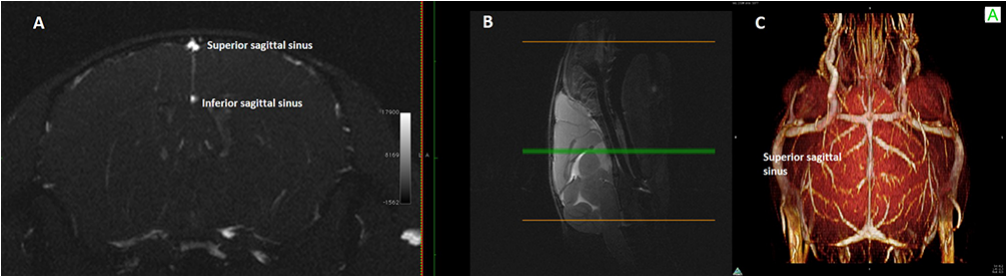
Single axial magnetic resonance angiography partition and 3D rendering of mouse head. **(A)**Single axial magnetic resonance angiography partition at the mid-level of the brain hemispheres (**(B)** mid-sagittal T2w slice used as a reference) and **(C)** 3D rendering of magnetic resonance angiography acquisition of a mouse head, as seen from above, The position of the superior sagittal sinus and inferior sagittal sinus is clearly shown as well as the connections of the former on the dorsal surface of the brain.

**Fig 5:**
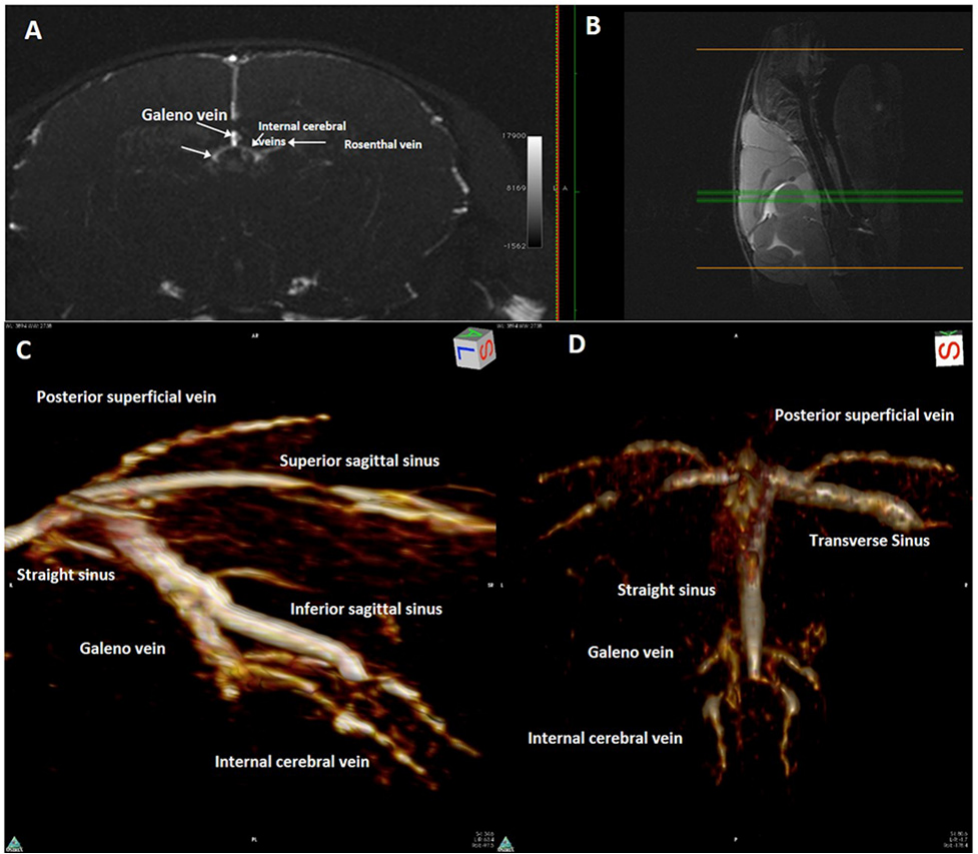
Magnetic resonance angiography and 3D of the caudal part of confluence of sinuses, and the deep brain venous system. **(A)** Maximum Intensity Projection of axial magnetic resonance angiography partitions at the mid-level of the brain hemispheres (**(B)**mid-sagittal T2w slice used as a reference,) and 3D rendering of magnetic resonance angiography acquisition of the caudal part of a mouse head, (**(C)**lateral view; **(D)** view from above), The confluence of sinuses, and the deep brain venous system (internal cerebral veins, Galeno vein, straight sinus, inferior sagittal sinus) can be easily appreciated as it resembles the configuration in humans.

**Fig 6:**
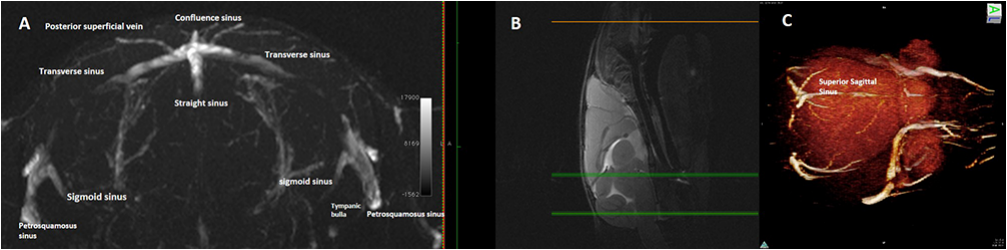
Magnetic resonance angiography with 3D of the confluence of sinuses and connections of the transverse sinuses. **(A)** Maximum Intensity Projection of axial Magnetic resonance angiography partitions, **(B)** at the occipito-cerebellar border (mid-sagittal T2w slice used as a reference), and **(C)** 3D rendering of magnetic resonance angiography acquisition of that region as seen from above. The confluence of sinuses and the connections of the Transverse sinuses are depicted.

The SSS starts anteriorly (Fig 4) and drains the dorsal and lateral parts of the frontal and parietal areas. The vessel originates behind the junction of the olfactory bulb and the frontal lobe, courses along the curvature of the inner table of the skull, in the interhemispheric sulcus, to reach the confluence of sinuses. In its course, the sinus is interconnected supraorbitally with the rostral rhinal veins, branches of superficial temporal vein and facial vein, and receives venous tributaries of the superficial venous system (anterior superficial cerebral vein). The SSS drains into the confluence sinuum together with the straight sinus (Figs 5, 6A and 6B).

**Fig 7:**
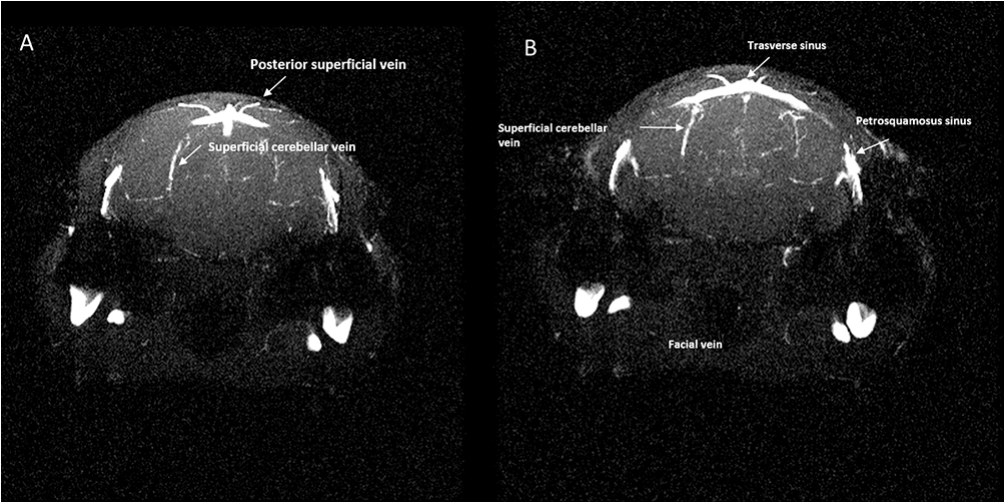
Magnetic resonance angiography of the transverse sinus. **(A)**, **(B)**, Single axial magnetic resonance angiography partitions at two levels showing **(A)** the tributaries of the transverse sinus and its lateral bifurcation into the straight sinus and **(B)** petrosquamosal sinus.

Veins that lead directly into this confluence drain a portion of the occipital cortex (Fig 7).

The Inferior Sagittal Sinus (ISS) begins on the midline in the central part of the brain, runs parallel to the SSS deeper in the interhemispheric sulcus and joins caudally the Galeno vein (GV) (Figs 4A and 5C) to form the straight sinus.

The TS, running along the posterior border the tentorium, receives the superior petrosal sinus, the posterior superficial cerebral vein and the superficial cerebellar veins (Figs 6 and 7).

**Fig 8:**
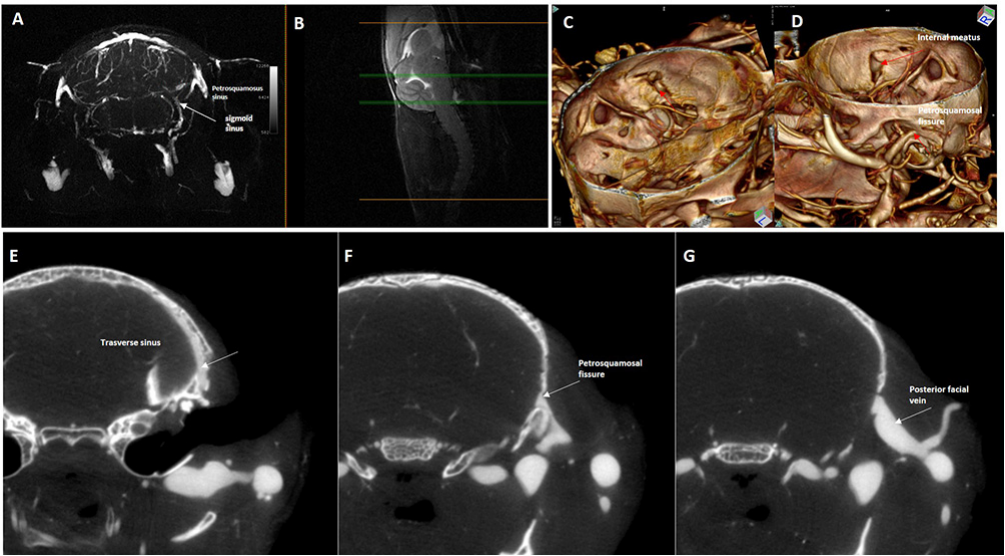
Magnetic resonance angiography with 3D rendering and silicon-computed tomography angiography of the petrosquamosal fissure. Upper row: Maximum Intensity Projection of axial magnetic resonance angiography partitions (slab at the occipito-cerebellar border **(B)** mid-sagittal T2w slice used as a reference,) and 3D rendering of silicon-enhanced computed tomography angiography as seen from **(C)** inside and **(D)** outside of the skull. **(E)**, **(F)**, **(G)** Lower row: Axial computed tomography slices in caudo-cranial sequence showing the exit from the brain of most of the transverse sinus blood through the petrosquamosal fissure (arrows), then joining the external jugular system.

The TSs continue caudally and ventrally, on each side, near the petrosquamosal fissure, each of them bifurcating into two branches: the sigmoid sinus (SS) and the petrosquamosal sinus (Fig 8).

The petrosquamosal sinus crosses the cranial bone, curves ventrally around the external margin of the timpanic bulla, and then emerges through the wide petrosquamosal fissure (“spurious” Jugular foramen), to run extracranially between the spurious jugular foramen and the temporomandibular joint and external auditory meatus (Fig 8E–8G). It divides into a branch that drains into the posterior facial vein and a branch that drains into the maxillary vein (Fig 8G). The micro-CT clearly demonstrated the relationship between the intracranial and extracranial veins with connection through three cranial foramina (Fig 8A–8G).

Intracranially, the TS continues in the SS that is a thin, atretic branch that runs dorsally around the medial border of tympanic bulla (Fig 6). The SS also receives the inferior petrosal sinuses and veins coming from the lateral aspect of pons and medulla and shows anastomoses with the vertebral venous plexus (Fig 8A), then crosses the jugular foramen, in the IJV.

While running along the inner table of the posterior fossa, the TS receives the superior petrosal sinus, the caudal rhinal veins and the cerebellar veins (Fig 7A).

As in humans, the deep system is composed by the internal cerebral veins (ICV), the basal vein, the Galeno vein (GV) and their tributaries.

The GV (Fig 5) runs posteriorly along the interior–midline of the cerebrum, and receives the ICV together with the Rosenthal veins.

The straight sinus drains the deep venous system through the GV as well as much of superficial venous system indirectly through the ISS. Posteriorly, the straight sinus empties into confluence sinuum (Fig 5), as in humans.

The VVs collect the blood of the district of the midbrain, cerebellum and occipital plexus, and run together with the corresponding artery in the transverse foramen. The posterior cranial fossa and cerebellum veins are also drained by the vertebral venous plexus.

### Extra-cerebral and neck veins

**Fig 9:**
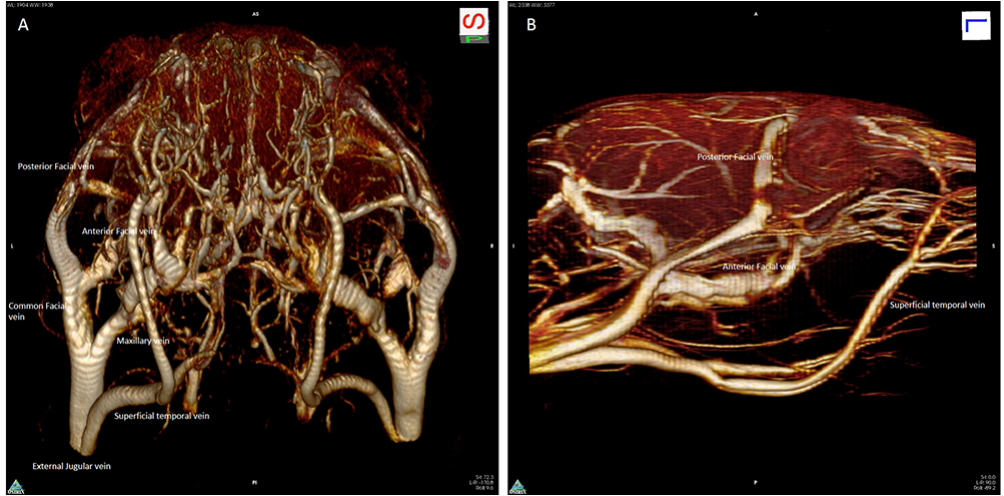
3D magnetic resonance angiography of external jugular vein. 3D magnetic resonance angiography rendering of the extracranial vasculature as seen **(A)** in a frontal-inferior view and **(B)** in a lateral view. The main facial venous trunks are well-shown, running caudally to join together to form the external jugular vein.

The major venous output from the head is the EJV, which is formed at the junction of the posterior facial vein, the anterior facial vein, the maxillary vein and the superficial temporal vein on each side of the head (Fig 9). This vein then runs ventral to the clavicle into the thorax, converging with the internal jugular and subclavian veins into the ipsilateral superior vena cava.

**Fig 10:**
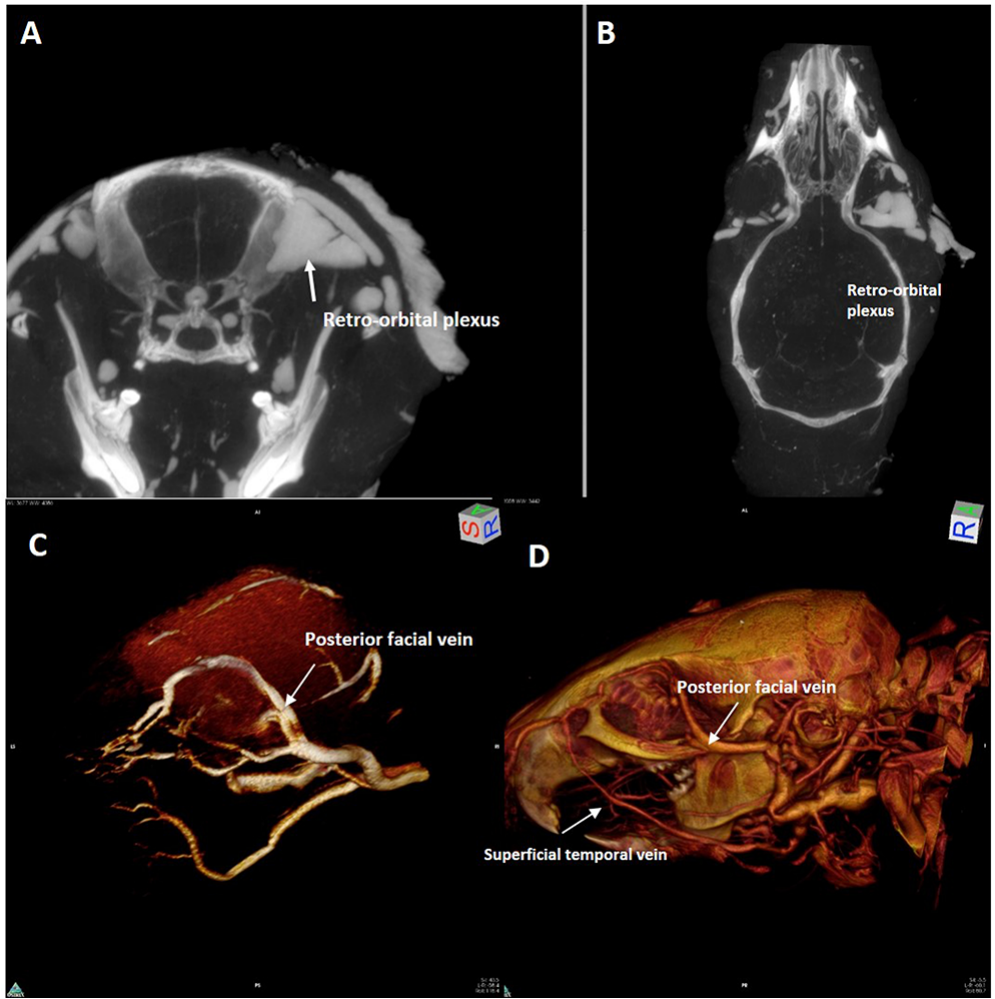
Magnetic resonance angiography with 3D rendering acquisition of the orbital region. Upper row: **(A)** axial and **(B)** coronal computed tomography slice. Lower row: 3D rendering of magnetic resonance angiography acquisition of the left orbital region (**(C)** antero-lateral view,). 3D rendering of silicon-enhanced computed tomography angiography (**(D)**lateral view,).**(A)**, **(B)**,The large retro-orbital venous plexus (white arrow) is clearly visible as well its drainage into the posterior facial vein (**(C)**, **(D)** white arrow).**(D)**The superficial temporal vein is also shown in.

Posterior to the ocular bulb there is a large venous plexus (Fig 10A and 10B) that flows laterally in the posterior facial vein (Fig 10C and 10D).

**Fig 11:**
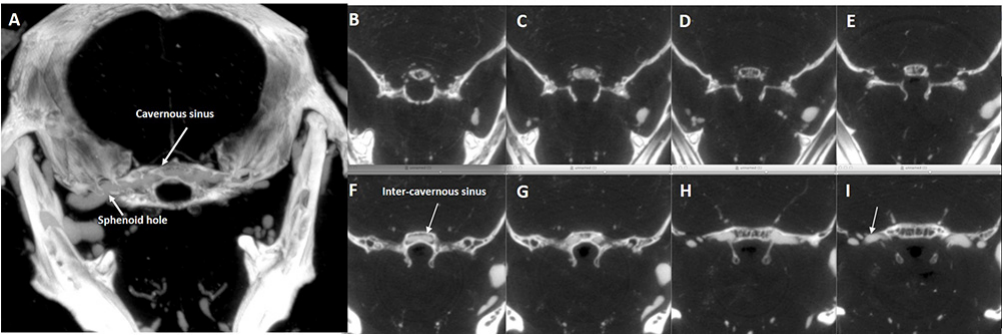
Axial computed tomography of the middle cranial fossa. Axial computed tomography slices at the level of the middle cranial fossa of the basicranium; **(A)** = 1mm-thick “MIP-ped” slab; **(B)**-**(I)** consecutive 0.05mm-thick slices in a cranio-caudal sequence showing the confluence of small vessels in the hypothalamic region **(B)**, **(C)**, **(D)** toward a large intraosseus median inter-cavernous sinus **(E)**, **(F)** which bilaterally exits the brain **(G)**, **(H)** to join the external jugular vein system (arrow in **(I)**).

Small venous branches of the ophthalmic plexus run medially toward the region of the cavernous sinus. The cavernous sinus forms medially a large intercavernous sinus (Fig 11).

The cavernous sinus drains into the superior petrosal sinus, which is divided into two branches, the largest crossing the cranial bone at the level of sphenoid bone (Fig 11H and 11I) and draining into the branches of the EJV, the smallest flowing downward into the SS. The cavernous sinus drains laterally along superior petrosal sinus to EJV and medially along the inferior petrosal sinus into the sigmoid-TS complex.

**Fig 12:**
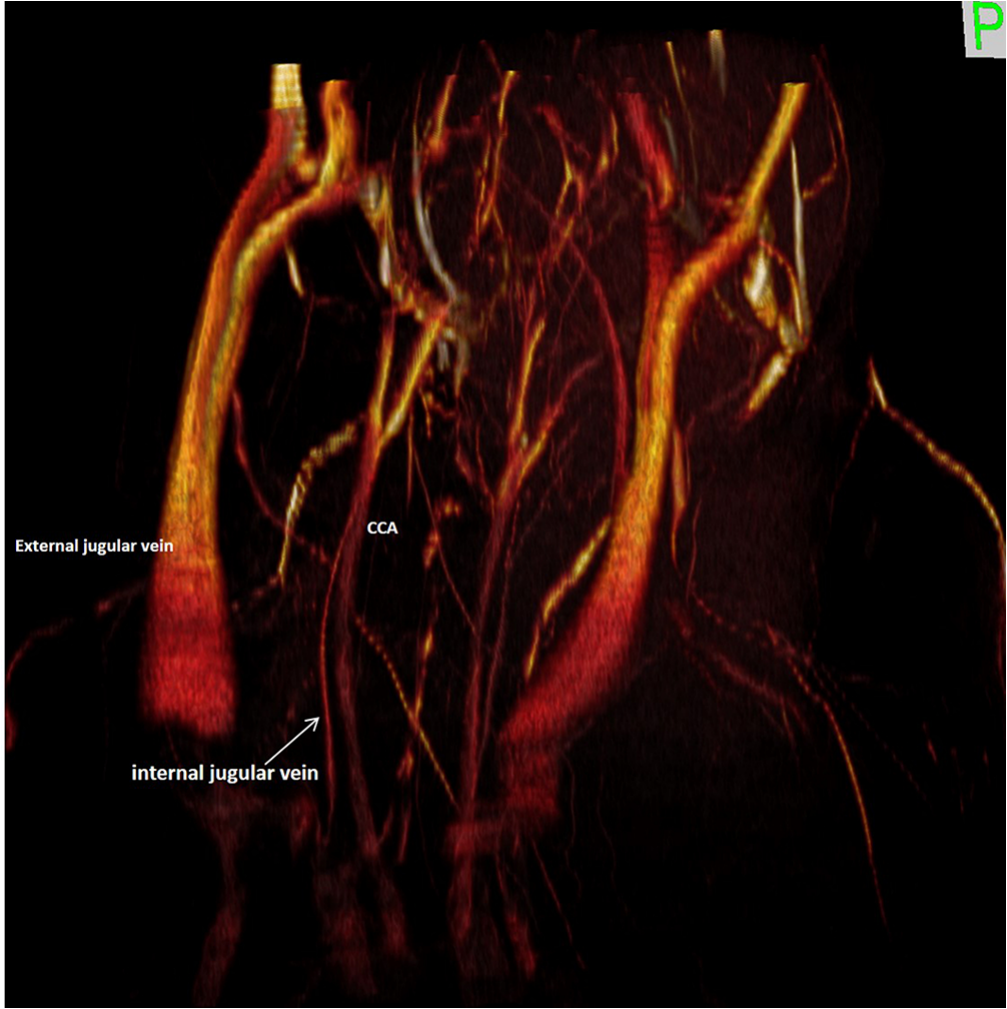
Magnetic resonance angiography with 3D rendering of the neck vessels. 3D rendering of the magnetic resonance angiography acquisition of the neck vessels (right anterior oblique view), showing the external jugular vein, the internal jugular vein and the common carotid artery.

**Fig 13:**
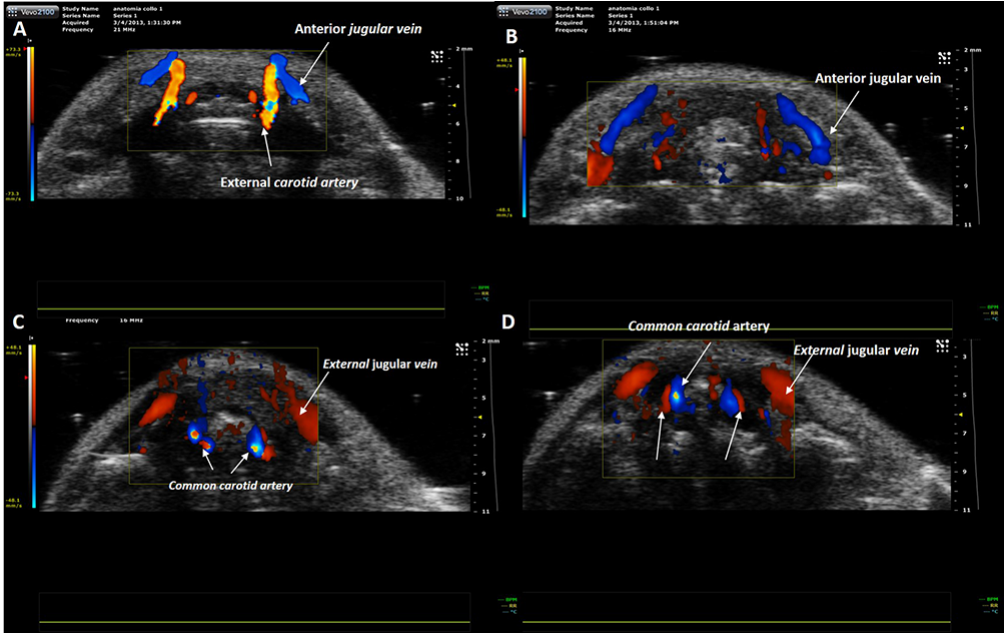
Eco-color Doppler examination. Craniocaudal scans performed with transverse planes of the upper and middle third of the mouse neck. **(A)** At the level of the upper third of the neck the external carotid artery runs anteriorly. **(B-D)** At level of middle third of the neck arterial and venous vessels are detectable. **(B)** The blood flow of anterior jugular veins is directed toward the external jugular veins. **(C-D)** The internal jugular veins are thin and in the typical position, anteriorly and laterally to the carotid artery.

**Fig 14:**
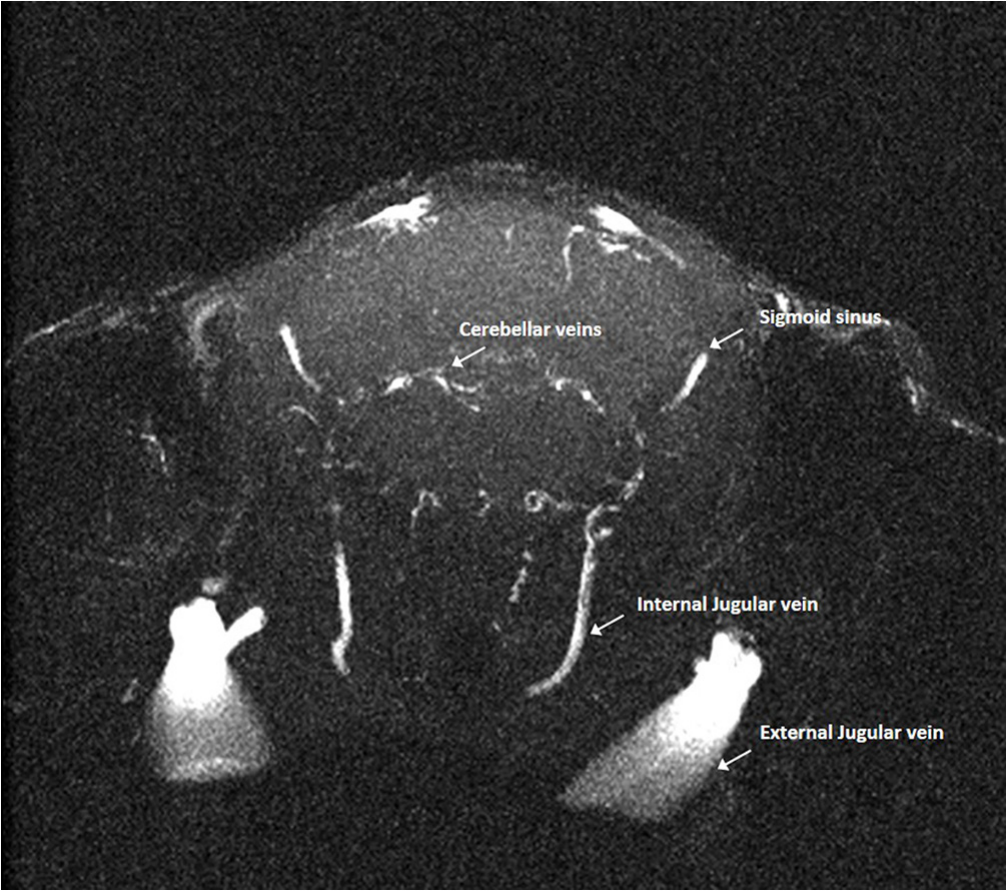
Magnetic resonance angiography of the internal jugular veins. Axial magnetic resonance angiography partitions at the level of the posterior cranial fossa showing some tributaries (e.g. cerebellar veins) of the internal jugular veins, which, differently from humans, is clearly hypotrophic in respect to the external jugular vein.

**Fig 15:**
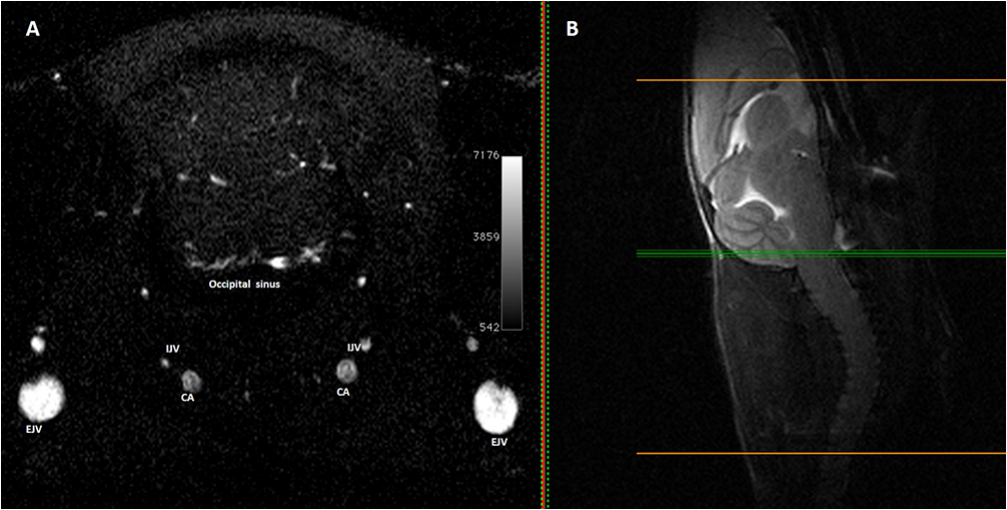
Magnetic resonance angiography of the lower part of the cerebellum and occipital plexus. **(A)** Single axial Magnetic resonance angiography partition at the level of the lower part of the cerebellum (**(B)** mid-sagittal T2w slice used as a reference) showing the occipital plexus. V: External Jugular Vein, IJV: Internal Jugular Vein, CA: Carotid Artery.

The thin IJVs run in a typical position, anterior and lateral to the Carotid Artery (Figs 12 and 13), and drain the SSes (Fig 14), the occipital plexus (Fig 15) of the posterior cranial fossa and the cerebellar veins.

**Fig 16:**
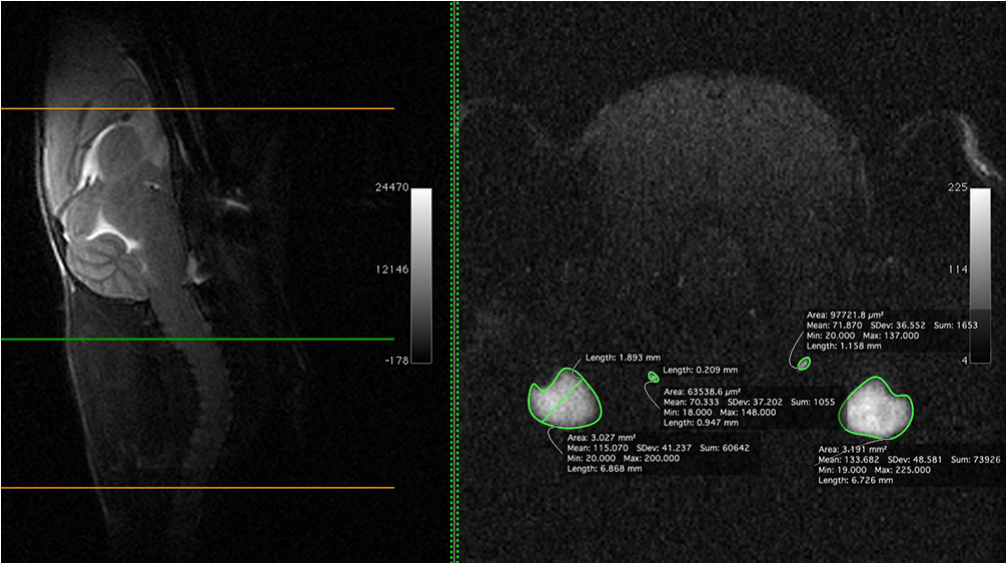
Magnetic resonance angiography of external jugular vein and internal jugular vein with size measurements. **(B)** Single axial magnetic resonance angiography partition at the middle level of the neck (**(A)** mid-sagittal T2w slice used as a reference,) showing the relative size (area, diameter, etc) of the external jugular vein and internal jugular vein.

**Fig 17:**
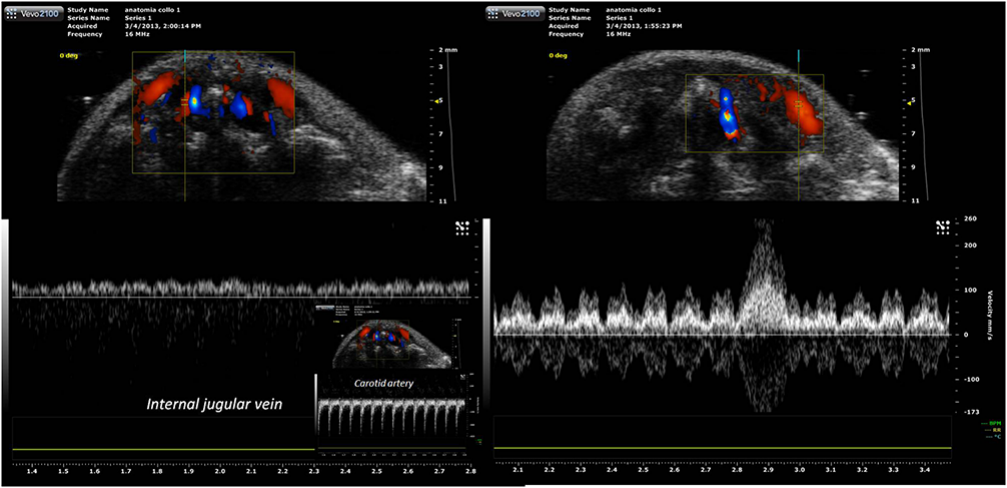
Eco-color doppler with pulsed doppler analysis of veins. **(A)**The internal jugular vein waveform is characterized by a monophasic pattern and low pulsatility index. **(B)**The external jugular vein is characterized by three-phasic pattern with high pulsatility. **(C)** In the small inset on the left the spectral analysis of the carotid artery blood flow is shown.

EJV, IJV, and VVs have been insonated at different depths. The largest vein in the cervical region was the EJV that collects blood from the major part of the neck (Fig 16). The mean IJV diameter was 0.2 mm (200 μm) at the middle level of the neck, with a mean area of 0.08 mm^2^, while the mean diameter and area for the EJV at the same level were 1.9 mm and 3 mm^2^, respectively (Fig 16). The vein is not easily compressed; it is in a deep position, spaced from the surface by a thick layer of neck muscles. The cerebral blood flow appears to drain primarily into the EJV and VV and IJV and the flow could be collateralized. The waveform from different veins are presented in Fig 17A and 17B.

The IJV is characterized by a low pulsatility waveform with a monophasic pattern and low pulsatility index. Conversely, EJV is characterized by three phasic pattern with high pulsatility, a small backflow component, resulting in high Pulsatility Index.

**Fig 18:**
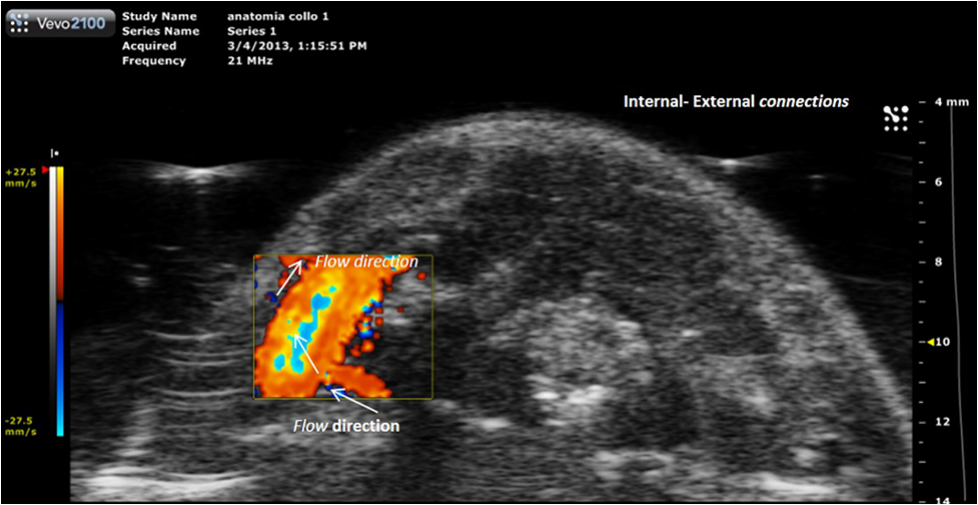
Eco-Color Doppler of vein collaterals. Collateral vein that connect external jugular vein and vertebral plexus. The flow is directed from vertebral plexus to the right external jugular vein.

Many collateral veins with anterior-posterior direction that were not identified in MRI and micro silicon-TC, were visible at Eco Color Doppler (ECD) (Fig 18).

## Discussion

The precise anatomical structure of the cranial venous outflow has been described using US, micro-CT and MRA techniques with regard to the connections between intra and extracranial veins. Our observations on the mice anatomy of the cerebral veins confirm and more extensively describe the previous findings in rabbit and rats [19–23]. We have demonstrated the presence of a communication between facial veins, orbital veins and the cavernous system. As this interconnection system is valveless, blood can flow in any direction either to or from the brain.

While animal models are invaluable research tools in helping us understand human diseases, in case of the cerebral venous system they are imperfect models for studying the human cerebral venous circulation. In mice the neck veins are always at the same level of the heart and, therefore, they are vessels with non- varying resistance, always open and constantly representing the major cerebral outflow pathway. Instead, in humans, the positioning of neck veins above heart level in upright position causes them to collapse, leading to an increased outflow resistance and to a cerebral outflow pathway that occurs predominantly through the vertebral venous plexus [24].

It may be speculated that the intra-extracranial venous connections that we have demonstrated in the mouse could also represent a possible drainage pathway in the upright position in humans, partly explaining the remaining venous flow “mismatch” observed by Valdueza et al in 23 young healthy adults studied in supine and upright position by colour-coded duplex sonography, in whom the drop in jugular flow in the upright position was not adequately compensated by an increase in vertebral vein flow [25]. The pattern of the dorsal cranial venous system and deep venous system of the mouse is quite similar to the human (SSS, Rosenthal vein, GV, straight sinus, TSs and confluens sinuum). The TS runs between the temporo-mandibular joint and the external auditory meatus and drains into extracranial veins at the base of the skull. The SS drains in IJV, that is a tiny vessel and receives tributaries from cerebellum, occipital lobe and midbrain.

The cavernous sinus passes through a foramen of the sphenoid bone, to join with the EJV. This demonstrates that the intracranial blood is predominantly drained by the extracranial district also for the drainage of cavernous sinus.

The intracranial venous outflow runs in three directions: into the IJV and EJVs and into the vertebral venous plexus.

**Fig 19:**
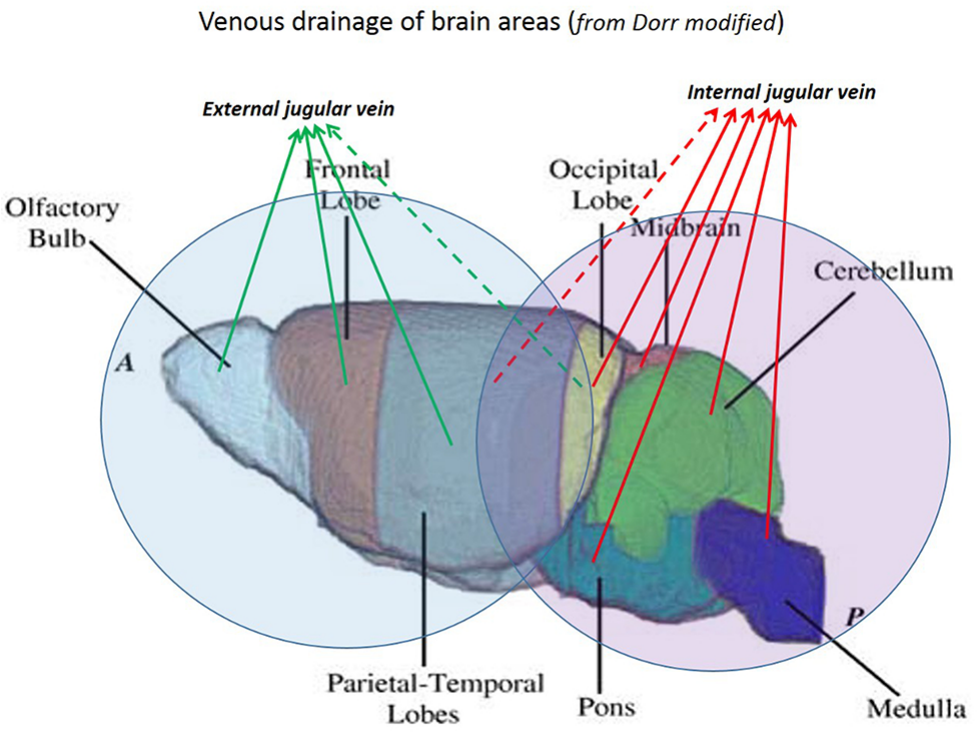
Cerebral areas with different venous drainage. Brain areas can be identified and divided according to the dominant venous drainage. The olfactory bulbs and frontal and parieto-temporal lobes drain mainly in the external jugular veins. The occipital lobe and cerebellum drain mainly in the internal jugular veins (modified from Dorr 2007 [27]).

Part of the venous drainage of the brain ultimately exits via the TS that is connected both with EJV and IJV. Unlike humans, the EJVs predominate on the IJVs. The venous drainage of the mouse brain can be divided into two areas (Fig 19).

Therefore, the interruption of EJV does not allow complete interruption of cerebral drainage with many possibilities of extra-intracranial collaterals.

In a previous study, Atkinson et al [26] worked on establishing whether the EJV bilateral ligation can change the cerebral blood flow. They did not see any change in blood-brain barrier permeability, neuroinflammation, demyelination or clinical signs in Jugular Vein ligation compared to the changes in sham group. In our opinion, the use of mice as a vascular model for chronic venous hypertension is not plausible using a single vessel ligature.

The use of mice vein ligature as animal model for cerebral venous stasis to understand the associated pathophysiology requires careful evaluation of mice vasculature and precise occlusion techniques, in order to successfully obstruct blood drainage from the brain.

A large amount of blood flow is collected in the EJVs draining from the olfactory bulbs and from ocular tissues. Moreover, the venous system contains connections between routes with potential collateralization. Therefore, if EJVs are occluded at the neck level many collateral pathways can compensate the venous hypertension.

The use of mouse as a vascular model for chronic venous hypertension in humans is not plausible, unless it is demonstrated that no other drainage pathways are activated.

Moreover, it is interesting to note that, at MRA, the SSS appeared thinner in its central part and wider both at its cranial and caudal end (i.e. at the connection with the rostral rhinal veins and at the confluence of sinuses, Fig 4C). This morphology suggest a possible bi-direction flow along its course, the blood flow being directed forward in the anterior part and backward in the posterior part. We thus assumed that, as in humans, the intracranial venous flow follows the large-scale gradient of vessel caliber. At the level of the neck veins, the flow direction was assessed by means of US color Doppler and showed the expected cranio-caudal direction.

Among the limitations of the present work, one should consider that the injection/infusion of the silicon gel for CT angiography might lead to pathological deformation of the vascular system. In addition, the desirable saturation of arterial flow in the MRA acquisition of the venous system may lead to unwanted obscuration of the signal from venous tracts where flow speed and direction changes abruptly.

We have provided a description of the cerebral venous drainage system of the mouse brain and neck. We identified many of the major branches and our result could be useful for future studies involving mouse cerebral veins. There are clear vascular differences between mouse and human anatomy. Three connections between intracranial and extracranial veins seem to be important drainage pathways of cerebral circulation in mouse and are absent in the humans were identified:

1. the petrosquamous sinus, already described in previous studies, that drains into the posterior facial vein.
2. the veins of the olfactory bulb, that drain in the superficial temporal vein through a foramen of the frontal bone.
3. the cavernous sinus, that drains in the EJV through a foramen of the sphenoid bone.
